# Urea Unfolding Study of *E. coli* Alanyl-tRNA Synthetase and Its Monomeric Variants Proves the Role of C-Terminal Domain in Stability

**DOI:** 10.1155/2015/805681

**Published:** 2015-11-04

**Authors:** Baisakhi Banerjee, Rajat Banerjee

**Affiliations:** Department of Biotechnology and Dr. B. C. Guha Centre for Genetic Engineering and Biotechnology, University of Calcutta 35, Ballygunge Circular Road, Kolkata 700 019, India

## Abstract

*E. coli* alanyl-tRNA exists as a dimer in its native form and the C-terminal coiled-coil part plays an important role in the dimerization process. The truncated N-terminal containing the first 700 amino acids (1–700) forms a monomeric variant possessing similar aminoacylation activity like wild type. A point mutation in the C-terminal domain (G674D) also produces a monomeric variant with a fivefold reduced aminoacylation activity compared to the wild type enzyme. Urea induced denaturation of these monomeric mutants along with another alaRS variant (N461 alaRS) was studied together with the full-length enzyme using various spectroscopic techniques such as intrinsic tryptophan fluorescence, 1-anilino-8-naphthalene-sulfonic acid binding, near- and far-UV circular dichroism, and analytical ultracentrifugation. Aminoacylation activity assay after refolding from denatured state revealed that the monomeric mutants studied here were unable to regain their activity, whereas the dimeric full-length alaRS gets back similar activity as the native enzyme. This study indicates that dimerization is one of the key regulatory factors that is important in the proper folding and stability of *E. coli* alaRS.

## 1. Introduction

Aminoacyl-tRNA synthetases (aaRSs) belong to a group of enzymes that are important in the protein biosynthesis process. They covalently link an amino acid to its cognate tRNA molecule containing the triplet anticodon specific for that amino acid. Despite catalyzing the similar kind of aminoacylation reaction, depending on the distinct active site topologies aaRSs have been classified into two unrelated classes: class I and class II [1–4]. In class I enzymes, the active site is formed by the Rossmann dinucleotide binding domain whereas in class II synthetases, a seven-stranded antiparallel *β*-fold is responsible for the active site formation [5].

*E. coli* alanyl-tRNA synthetase, class IIa enzyme has been previously described either as tetramer [6] or as dimer [7, 8]. Recently, based on analytical ultracentrifugation experiment, Dignam et al. have shown that this enzyme is a homodimer of 875 residues with molecular weight of 96,000 Da per subunits/monomer [9]. There are four functional domains that constitute the structure of this particular enzyme. The class IIa aminoacylation and tRNA recognition domain (N-terminal 1–461 amino acids) is responsible for the aminoacylation of tRNA and also activation of alanine. The editing domain (amino acid 553–705) hydrolyzes misactivated glytRNA^Ala^ and sertRNA^Ala^. The C-terminal oligomerisation domain (amino acid 705–875), which is responsible for the dimer formation, plays a role in the tRNA recognition [10]. Interestingly, this enzyme also has a unique transcriptional regulation mechanism [11]. It was demonstrated that alaRS binds the palindromic DNA sequence flanking its own gene's transcriptional start site and repress its own gene transcription [11]. It was also reported that the amount of repression is increased in the presence of elevated concentration of its cognate amino acid substrate, L-alanine [11].

Recently crystal structure of the full-length enzyme from* Archaeoglobus fulgidus* in the presence of tRNA was reported [12]. Moreover, the structure of the N-terminal catalytic fragment and C-terminal oligomerization domain of the same enzyme from* E. coli*,* Aquifex aeolicus*,* Pyrococcus horikoshii*, and* Archaeoglobus fulgidus* was also reported either in complex with its ligands or in absence of any [10, 13–17]. Previous reports showed that the N-terminal 461 amino acid residues are sufficient for the aminoacylation of tRNA^Ala^ [18]. Moreover, alaRS monomer containing the N-terminal 700 amino acids has almost the same *k*_cat_ value for tRNA^Ala^ aminoacylation when compared to the full-length dimeric (875 amino acids/monomer) protein [18]. In another study, it was shown that point mutation in the amino acid residue 674 (where the glycine has been changed to aspartic acid) results in a full-length monomer that has 20-fold reduction in the *k*_cat_ value for tRNA aminoacylation [19].

Keeping in mind the unusual features of this enzyme with large multidomain dimeric structure, in this study we have investigated how the C-terminal domain affects the stability of this enzyme. In this report, we have constructed three mutant proteins. The first mutant is a full-length monomer having a mutation in the 674 position (G674D, previously demonstrated to form full-length monomer, vide supra). The second mutant contains the N-terminal aminoacylation domain (1–700, N700). The third mutant has N-terminal 461 amino acids (1–461, N461), a minimum fragment required for the proper aminoacylation of tRNA^Ala^. These mutant proteins and the wild type enzyme were subjected to denaturation in the presence of urea to decipher the unfolding pathway of these proteins under equilibrium condition. This study reveals that WT-alaRS is more stable than the mutant alaRSs and also have the capacity to refold back from the fully denatured state unlike the other mutants studied here.

## 2. Materials and Methods

### 2.1. Materials

The clone of full-length* E. coli* alaRS (WT-alaRS), N700 alaRS, and plasmid containing tRNA^Ala^ transcript was a kind gift from Professor Paul Schimmel (Skaggs Institute for Chemical Biology, The Scripps Research Institute, California). All the chemicals that are used in this study are of analytical grade and were purchased from Sigma (St. Louis, MO), SRL (India), E-Merck (Germany), and HI-MEDIA (India).

### 2.2. Construction of* E. coli* N461 and G674D alaRS Mutant

For construction of N461 truncated enzyme and G674D variant, the primer 5′ATT TAA AGG CTA TGA CTA ACT GGA ACT GAA 3′ and 5′ AGCCGCA CTGATGATAT TGGT 3′ were used, respectively. Mutagenesis was carried out by using in vitro site-directed mutagenesis protocol by Stratagene and the mutation was confirmed by DNA sequencing.

### 2.3. Purification of* E. coli *Alanyl-tRNA Synthetases

Wild type alaRS, N461, and G674D alaRS with a N-terminal histidine tag were expressed in BL21 (DE3) after transformation of plasmid pET28a containing the desired constructs. The plasmid pET20a encoding N-terminal histidine tag N700 alaRS was also transformed in BL21 (DE3). Single colonies were picked in all cases, inoculated into 10 mL of Luria-Bertani (LB) medium containing 50 *μ*g/mL Kanamycin except N700 where 100 *μ*g/mL of ampicillin was added, and grown overnight at 37°C with constant shaking at 250 rpm. On the following morning, 1% of overnight culture was transferred to 1 L of LB medium and grown at 37°C until OD_600_ = 0.6; cells were then induced with 1 mM of IPTG and further grown at the same temperature for 4 hrs. The cells were pelleted by centrifugation at 5000 rpm for 15 min at 4°C. Except N700 alaRS, the pelleted cells were lysed with buffer A (50 mM Tris-HCl, pH 8.0 containing 500 mM NaCl, 10 mM imidazole, 10% glycerol, and 1 mM *β*-mercaptoethanol). For N700 alaRS, the buffer composition was 100 mM Tris-HCl, pH 8.0 containing 10% glycerol, and 1 mM *β*-mercaptoethanol. The suspended cells were sonicated and centrifuged at 25000 rpm for 1 h. The supernatant was loaded on an Ni-NTA agarose column (preequilibrated with buffer A). The column was then washed with 2 column volumes of buffer A; finally* E. coli* alaRS and its mutants were eluted with a gradient of imidazole concentration ranging from 20 to 200 mM in buffer A. All the eluted fractions were checked by 12% sodium dodecyl sulfate-polyacrylamide gel (SDS-PAGE) electrophoresis. The major eluted fractions containing desired proteins were pooled and dialyzed against buffer C (100 mM Tris-HCl, pH 8.0 containing 100 mM NaCl, and 20% glycerol) at 4°C. After 36–40 hrs of dialysis, samples were collected from the dialysis bag and protein concentration was determined by measuring absorbance at 280 nm using the molar extinction coefficient of the protein (71195 M^−1 ^cm^−1^ for WT-alaRS as a monomer and G674D, 65320 M^−1 ^cm^−1^ for N700, and 60580 M^−1 ^cm^−1^ for N461).

### 2.4. Fluorescence Spectroscopy

Fluorescence measurements were performed in a Hitachi F-7000 spectrofluorimeter with 1 mm quartz cuvette at 25°C. All the experiments were repeated at least 3 times. The fluorescence emission was recorded in the wavelength region of 310–450 nm after exciting the protein solution at 295 nm for selective observation of tryptophan residues. The bandpasses were 5 nm for both excitation and emission. The protein concentrations were 4 *μ*M. Proteins were incubated in the presence or absence of increasing concentration of urea (0–8 M) for 16–18 h at 25°C in 100 mM Tris-HCl, pH 8.0 buffer at room temperature before the spectra were recorded. Controls containing appropriate concentration of denaturants were prepared and were subtracted from the corresponding samples spectra. For the experiments, where ANS (30 *μ*M) had been used, the excitation wavelength was 420 nm to avoid inner filter effect and emission was recorded at 482 nm. Appropriate blanks were also subtracted from each experiment.

### 2.5. Circular Dichroism (CD) Spectroscopy

The CD spectra measurements were carried out in a JASCO J-815 spectropolarimeter, using 100 mM Tris-HCl (pH 8.0) buffer. The spectra were recorded at 25°C using 1 mm and 10 mm path length cuvettes for far-UV (210–260 nm) and near-UV (260–320 nm) CD measurement, respectively, with a scan speed 50 nm/min. The final spectrum was average of five independent measurements. For far-UV and near-UV CD experiments the protein concentrations were kept at 4 *μ*M and 15 *μ*M, respectively. The sample spectra were corrected by subtracting from the buffer spectra containing the same concentration of urea without the protein. The following equation was used to calculate the mean residue ellipticity (MRE) of the protein MRE = (100 ×  observed *θ* × MW)/(10 × *l* × *C* × *n*) in deg·cm^2^·dmol^−1^, where *θ* is the observed ellipticity in millidegrees, MW the molecular weight in kilodalton (kDa), *C* the concentration in mg/mL, *n* the number of amino acids, and *l* the path length in cm [20, 21].

### 2.6. Curve Fitting

Nonlinear least square fitting of the equilibrium denaturation data (both fluorescence intensity ratio and CD) was done according to the two following equations, corresponding to two-state and three-state models, respectively [22]:

(1) $$\begin{array}{c} S_{obs}=\frac{S_{N}+S_{U}e^{-\left(\Delta G_{1}-m_{1}\left[D\right]\right)/RT}}{1+e^{-\left(\Delta G_{1}-m_{1}\left[D\right]\right)/RT}}, \end{array}$$

where *S*_obs_ denotes observed intensity of spectroscopic signal, *S*_*N*_ and *S*_*U*_ denote the intensity of signals for the native and unfolded species, respectively, Δ*G* is the free energy of unfolding without denaturant, *m* is the slope of the transition, and concentration of the denaturant is *d*. Consider

(2) Sobs=SNSIe−ΔG1−m1D/RT+SUe−ΔG1−m1D/RTe−ΔG2−m2D/RT1+e−ΔG1−m1D/RT+e−ΔG1−m1D/RTe−ΔG2−m2D/RT.

In the above equation *S*_obs_ denotes intensity of the observed signal for spectroscopic data. *S*_*N*_, *S*_*I*_, and *S*_*U*_ denote the intensities of signal for the native, intermediate, and unfolded states, respectively. *R* represents the universal gas constant and *T* represents the temperature in absolute scale. Existence of one intermediate state is assumed while deriving the equation. It was also considered that Δ*G* varies linearly with denaturant concentration: Δ*G*_1_ = Δ*G*_*O*_ + *m*_*l*_[*D*]. There are five independent parameters in this equation such as Δ*G*_1_, the change in free energy between the native and the intermediate states, Δ*G*_2_, the change in free energy between the native and the unfolded states, *m*_1_, the slope of the first transition, *m*_2_, the slope of the second transition, and *S*_*I*_, the value of the spectroscopic parameter for the pure intermediate state. These parameters were floated globally and the fitting of data was done using software KyPlot (version 2.0 beta 15 (32 bits), Koichi Yoshioka, 1997–2001). Further the chi-square values (*χ* ^2^) were determined from the fitted data. The set of values that resulted in minimum (*χ* ^2^) was taken as the best fit. Data obtained from at least three independent experiments were used to calculate the average value.

### 2.7. Analytical Ultracentrifugation

Beckman Optima XL-I analytical ultracentrifuge containing absorbance optics was used to perform analytical ultracentrifugation experiments. The rotor used was An50Ti. Experiments were done at 20°C. Sedimentation velocity experiments were performed at 28,000 rpm in a charcoal-filled Epon double-sector centerpiece. Protein samples and the reference buffer solutions were centrifuged at 5000 rpm for 4 min at room temperature in a tabletop centrifuge to remove any suspended contaminants. Protein samples were maintained in 100 mM Tris-HCl, pH 8.0 buffer with or without the denaturant. Parameters like density and viscosity of the solvents were calculated using the program SEDNTERP [23]. The value of partial specific volume was found to be 0.7321 mLg^−1^ as calculated at experimental temperature, that is, 20°C from the sequence of amino acid using SEDNTERP. Before the collection of data, all the samples were allowed to equilibrate for 1 h under vacuum and temperature was allowed to get stabilised. Each centrifuged cell was scanned in a sequential manner by setting zero time delay between successive scans. Velocity data were collected at 280 nm at 0.003 cm intervals with one average in a continuous scan mode. Sedimentation velocity data were transferred to the program SEDFIT. All the data were analyzed with SEDFIT software (version 14.1, 2013) using the following specifications. A continuous *c*(*s*) model was used with a range of 0.5−12 S that had confidence level of 0.68 (1 standard deviation) [24]. Fitting was performed using alternating rounds of the simplex and Marquardt-Levenberg algorithms were employed for the fitting of data until no further decrease in root mean square deviation (RMSD) was observed. Samples were prepared 12 h prior to the experiment; protein concentrations were adjusted to 4 *μ*M. For sedimentation equilibrium experiments six-channel charcoal-filled epon centerpieces were used. Sedimentation equilibrium was attained at a rotor temperature of 20°C at different rotor speeds for different proteins for complete attainment of equilibrium. Absorbance profiles were obtained at a wavelength of 280 nm. Extinction coefficient ratios were estimated using spectrophotometry at different wavelengths. Further, 3–6 sedimentation equilibrium data sets that are obtained at different loading concentrations were analyzed by SEDPHAT (version 10.4, 2012) using global modelling [25]. During the fitting of data sets, molecular weight values were allowed to float. Molecular weights (Mr) were obtained with least chi-square and least RMSD value by a model-dependent method. Loading concentrations of alaRS proteins were kept at 5 *μ*M, 10 *μ*M, and 20 *μ*M. All the protein concentrations are that of the monomer.

### 2.8. Aminoacylation Assay

*E. coli *tRNA^Ala^ was purified as described in a previously published protocol [7]. All the alaRSs (protein concentration 4 *μ*M) were first unfolded by using different concentrations of denaturant as described in spectroscopic experiments. The assay were then started by rapidly diluting these denatured samples ten times with aminoacylation buffer (20 mM Hepes-NaOH (pH 7.5), 100 mM KCl, 0.2 mM EDTA, 0.2 mM L-alanine, 2 mM ATP, and 5 mM MgCl2) containing trace amount of L-[3-^3^H] Alanine (250 *μ*Ci, Perkin Elmer Lifesciences), 4 *μ*M* E. coli *tRNA^Ala^, and desired amount of denaturant. Aminoacylation was performed in 37°C in aminoacylation buffer. Ten microlitres of aliquots was removed at different time intervals. These aliquots were spotted on 3 MM filter paper disks (Whatman). Thereafter they were washed three times in 10% trichloroacetic acid and dried. The amount of radioactivity retained was determined by liquid scintillation; counting was employed to determine the amount of radioactivity retained in the disks. Acceptor activity was taken to be 50 nmol per mg of RNA.

## 3. Result

### 3.1. G674D, N700, and N461 alaRS All Forms Folded Monomer

**Figure 1:**
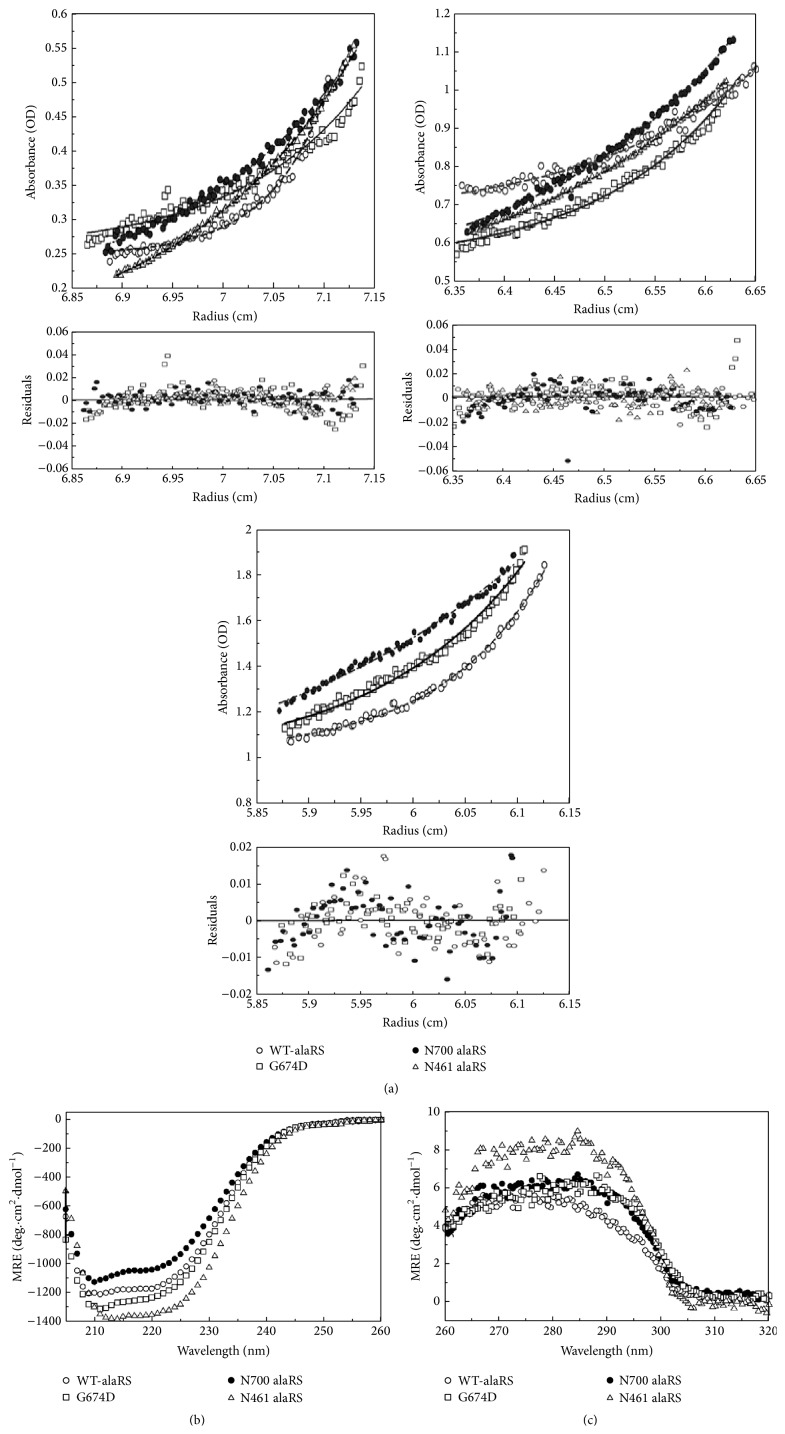
Biophysical characterization of mutant alaRSs along with WT protein. (a) Molecular weight determination of WT-alaRS, G674D, N700 alaRS, and N461 alaRS by analytical ultracentrifugation (AUC). Sedimentation equilibrium profiles of WT-alaRS and its mutants at concentrations 5 *μ*M (upper left panel), 10 *μ*M (upper right panel), and 20 *μ*M (lower panel) at 20°C. The buffer was 100 mM Tris-HCl, pH 8.0. Fitting of the data was performed using SEDPHAT software. (b) Far-UV CD and (c) near-UV CD spectra of WT-alaRS, G674D, N700 alaRS, and N461 alaRS. The result has been represented as mean residue ellipticity (MRE) at 25°C. Protein concentrations were kept at 4 *μ*M for far-UV CD and 15 *μ*M for near-UV CD with the same buffer as the analytical ultracentrifugation experiment. Path length of the cuvettes was 1 mm and 10 mm for far-UV CD and near-UV CD, respectively. Scan speed was 50 nm/min and five independent scans were performed and from that average spectra were taken.

Previous report indicated that the wild type* E. coli* alaRS was a dimeric enzyme of identical subunits [9]. It was also reported that the Gly to Asp mutation in the 674 position of* E. coli* alaRS results in a protein which forms a full-length monomer [19]. To reestablish the oligomeric nature of wild type alaRS (WT-alaRS), G674D alaRS along with N700 alaRS and N461 alaRS sedimentation equilibrium experiments had been done (Figure 1(a)). The N461 mutant protein formed aggregates at concentrations higher than 10 *μ*M. Fitting the data in SEDPHAT using a single species model yielded a good fit with a molecular weight ranging from 186280 to 197770 Da for WT-alaRS (theoretical molecular weight calculated from amino acid composition, 192500 Da), 92932 to 95331 Da for G674D (theoretical molecular weight, 96250 Da), 65971 to 72843 Da for N700 alaRS (theoretical molecular weight, 77000 Da), and 44601 to 50000 Da for N461 alaRS (theoretical molecular weight, 50710 Da). The theoretical molecular weight was calculated from amino acid composition. Thus, under our experimental conditions, the proteins exist as monomeric or dimeric states as reported earlier [9, 18, 19].

Circular dichroism proved to be a very useful indicator of the overall secondary and tertiary structure of a particular protein [21, 26]. The far-UV CD spectrum (Figure 1(b)) showed a well-folded secondary structure for WT-alaRS, G674D, N700, and N461 mutant proteins. The near-UV CD spectrum showed that N700 and G674D have almost similar kind of tertiary interaction that differs from the full-length enzyme. These two mutant proteins have an increased intensity in the region of 285–305 nm (that arises due to the tryptophan residue in a protein) than WT-alaRS. This could be due to the different folding pattern of monomeric mutants. In contrast, N461 showed a near-UV CD spectrum that is different from full-length as well as N700 and G674D alaRS (Figure 1(c)). This suggests that the tertiary interactions in these three mutant variants might be different from the full-length enzyme.

### 3.2. Unfolding of Wild Type alaRS and Its Mutants Is a Multistep Process

**Figure 2:**
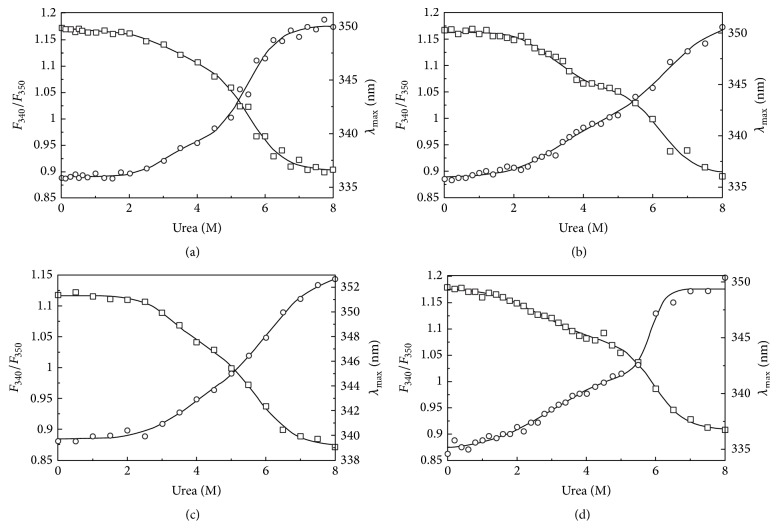
Unfolding profile of WT-alaRS (a), G674D (b), N700 alaRS (c), and N461 (d). The graph was made by plotting the fluorescence intensity ratio change (*F*_340_/*F*_350_) and alterations in emission maximum with increasing urea concentration. All the protein concentrations were kept at 4 *μ*M. Samples were excited at 295 nm where excitation and emission bandpasses were kept as 5 nm for both. A circulating water bath was used to maintain the temperature at 25°C. The buffer was 100 mM Tris-HCl, pH 8.0.

Many studies used intrinsic tryptophan fluorescence to investigate conformational changes in proteins [27, 28].* E. coli* alaRS has a total of eight tryptophan residues. Seven of them are present in the N-terminal half of the protein (positions 112, 120, 129, 143, 171, 194, and 212). The remaining tryptophan residue is present in the C-terminal domain (position 870). We examined the fluorescence emission spectrum of the wild type and three mutant alaRSs at different urea concentrations. At 0 M urea WT-alaRS showed an emission maximum at 336 nm, which is consistent with our previous study [29]. With increasing urea concentration the emission maximum was red-shifted and finally levelled off around 350 nm at 8 M urea. In contrast, N700 alaRS showed an emission maximum closer to 339 nm whereas the other two mutants N461 (335 nm) and G674D (336 nm) showed an emission maximum similar to the wild type full-length enzyme. At 8 M urea, red shifted emission maximum indicated a complete exposure of the buried tryptophan residues (Figure 2). The denaturation curve for all the alaRSs including wild type alaRS when plotted against the denaturation concentration showed two transitions with an indication of probable intermediate formation. Thermodynamic parameters (Table 1) were calculated for all the four alaRS proteins using (2) (for three-state model) as described in Section 2.

### 3.3. The Intermediate in the Urea Induced Unfolding Pathway Contains Exposed Hydrophobic Surfaces

**Figure 3:**
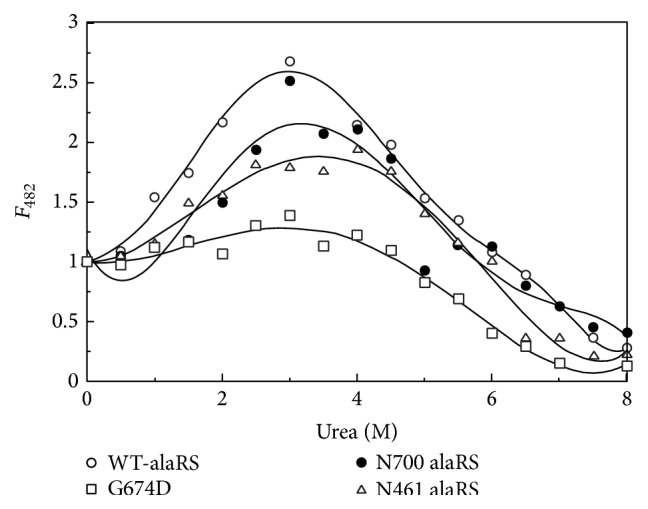
Change in normalized ANS fluorescence intensity with increased urea concentration. ANS and alaRS concentrations were 30 *μ*M and 4 *μ*M, respectively. Excitation wavelength was 420 nm and the bandpasses for both the excitation and emission were kept at 5 nm. The spectra were measured at 25°C. The samples were kept at dark for 1 h after the ANS addition. The values at 482 nm were plotted after they were normalized considering the 0 M values as 1.

ANS has been widely used as a marker to locate exposed hydrophobic patches on protein surface [30]. Intrinsic tryptophan fluorescence measurement indicated the presence of an intermediate in the urea unfolding pathway of all the alaRS proteins. To understand this intermediate forming phenomenon in detail, ANS binding experiments were carried out. To understand the ANS saturation of all the alaRS proteins a separate titration experiment was done. The result indicates that the dissociation constant for ANS binding for all the proteins falls under the 1–5 *μ*M range (data not shown). Depending on this result 30 *μ*M ANS (as a final concentration) was added to alaRS proteins with increasing urea concentration (Figure 3). For the WT-alaRS, ANS fluorescence gradually increased and reached a maximum at around 3 M urea and then decreased slowly and levelled off at 8 M of urea concentration. A similar increase of ANS fluorescence was also observed for N700 and in N461 alaRS, but the change was negligible for G674D alaRS (Figure 3). Taken together, these results suggested that there might be accumulation of an intermediate species during denaturation of WT and N700 and N461 alaRS that could bind ANS strongly. The increase in the ANS fluorescence intensity might result from either increased quantum yield or increased binding stoichiometry. As the emission maxima did not alter much after ANS binding in WT-alaRS, N700, and N461 alaRS, the observed enhanced fluorescence intensity was primarily due to increased binding sites (data not shown).

### 3.4. Far-UV CD Reveals Disrupted Secondary Structure of the Intermediate

**Figure 4:**
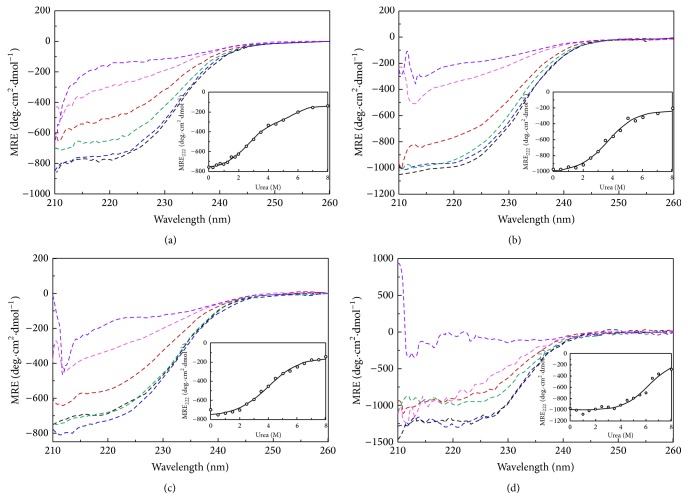
Far-UV CD spectra of WT-alaRS (a), G674D (b), N700 alaRS (c), and N461 alaRS (d) (4 *μ*M) using native protein (black line) and different concentrations of urea such as 1 M (blue line), 2 M (green line), 3 M (red line), 5 M (purple line), and 8 M urea (violet line). The temperature was at 25°C and the buffer composition was 100 mM Tris-HCl, pH 8.0. Inset shows the changes in the mean residue ellipticity (MRE_222_) as a function of increasing concentration of urea. The solid line represents the global fit. The cuvette used has 1 mm path length and the scan speed was 50 nm/min. The final spectra were an average of five independent scanned spectra. All of the spectra were subtracted from blanks containing appropriate concentration of denaturant.

To observe the effect of urea on the secondary structure, far-UV CD experiments of all the alaRS proteins were performed. The CD spectrum profile of wild type enzyme was stable up to 1 M urea and then showed continuous denaturation from 2 to 8 M urea (Figure 4(a)), whereas the N700 and G674D mutants were stable up to 2 M urea; then it got denatured from 3 to 8 M urea (Figures 4(c) and 4(b), resp.). However, the N461 mutant is stable up to 1 M urea, shows an intermediate level of denaturation from 2 to 5 M urea, and then completely denatures above 5 M (Figure 4(d)). From the far-UV CD data it was quite clear that WT-alaRS loses its secondary structure with increasing urea concentration, whereas N700 and G674D alaRS retain their secondary structure till 3 M of urea concentration. This feature often suggests the presence of a folding intermediate. Interestingly when mean residual ellipticity at 222 nm (MRE_222_) was plotted against the denaturant concentration to understand the changes in the *α*-helix content of the proteins, WT-alaRS data fitted well with a three-state equation, whereas the G674D, N700, and N461 alaRSs followed a two-state model equation (1). The calculated thermodynamic parameters are represented in Table 2.

### 3.5. Only WT-alaRS Has the Capability to Refold Back to Its Native-Like Architecture and Retained Activity

**Figure 5:**
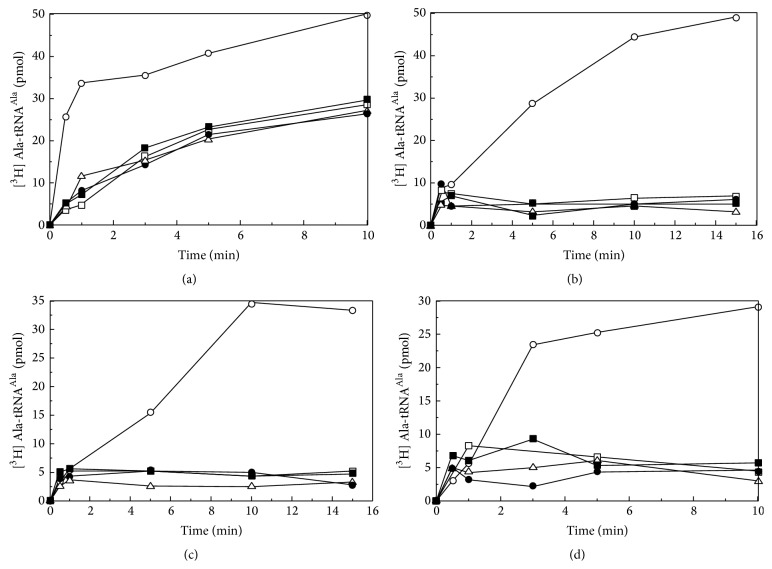
Aminoacylation assays of WT-alaRS (a), G674D (b), N700 alaRS (c), and N461 alaRS (d) were followed at 37°C for 10 min using native alaRS (open circle), overnight incubated denatured protein containing 0.3 M urea (filled square) and 0.8 M urea (open triangle), and ten times diluted protein samples from 3 M (open square) and 8 M (filled circle) of urea. Liquid scintillation counting was used to calculate the amount of radioactivity preserved. The concentrations of alaRS and tRNA were 400 nM and 4 *μ*M, respectively.

The aminoacylation assay was performed by rapidly diluting the overnight denatured protein samples with buffer. A complete reversibility of the enzyme activity was observed in case of wild type alaRS, whereas all the other three mutants were incapable of regaining back their activity when the samples were diluted from 8 M to 0.8 M or lower urea concentration (Figure 5).

### 3.6. Wild Type alaRS Intermediate Had Diminished Tertiary Interaction

**Figure 6:**
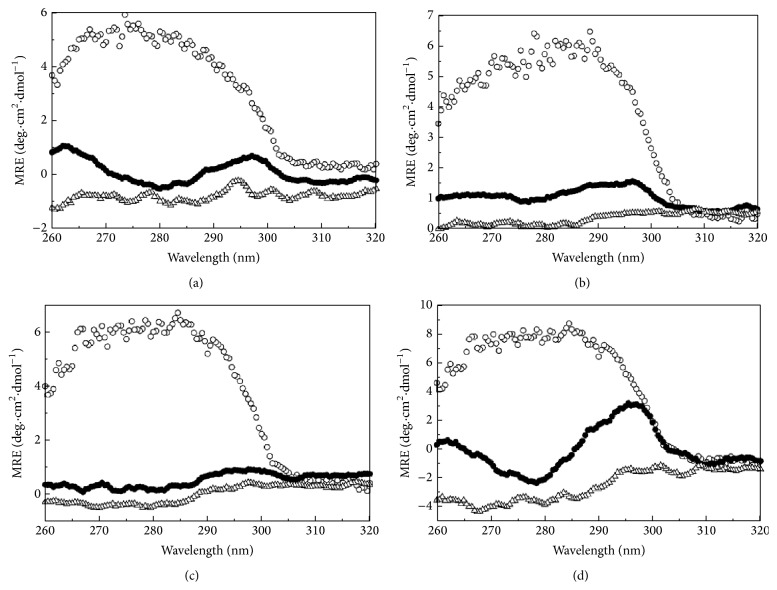
Near-UV CD spectra of WT-alaRS (a), G674D (b), N700 alaRS (c), and N461 alaRS (d) (15 *μ*M) without denaturant (open circle), in the presence of 3 M urea (filled circle) and 8 M urea (open triangle). The buffer composition was the same as the far-UV CD and the temperature was kept at 25°C. The experiment was recorded with a cuvette having a path length of 10 mm and 50 nm/min scan speed was maintained. Five independent spectra were averaged.

A large change in the ANS fluorescence intensity and retention of secondary structure were often observed in case of intermediates like molten globule [31]. Molten globule state of a protein was reported to have the following characteristics: wild-type-like secondary structure (invariant or little changed in far-UV CD spectrum, 200–250 nm), partially or completely diminished tertiary interactions (large change in near-UV CD spectrum, 260–320 nm), and increased number of exposed hydrophobic patches (increased ANS binding). Though WT and N700 alaRS had shown increased ANS binding, the secondary structure was lost as observed from the far-UV CD spectra (Figure 6), so the intermediate formed in this pathway might not be a molten globule-like intermediate. Changes in the tertiary structure can be deduced from CD study in the near-UV region (260–320 nm) where signal largely contributed to aromatic amino acids and cysteine [26]. To characterize the nature of the intermediate formed around 3 M of urea concentration near-UV CD experiments were carried out. The near-UV CD spectrum of WT and mutant alaRSs is shown in the presence of 0 M, 3 M, and 8 M of urea (Figure 6). In all the cases, the native protein (0 M urea) displayed a distinct positive side chain CD spectrum whereas in presence of 3 M as well as 8 M urea signal has completely disappeared indicating a complete loss of tertiary interaction.

### 3.7. Urea Induced Intermediate of Wild Type alaRS Is an Ensemble of Dimer and Monomer

**Figure 7:**
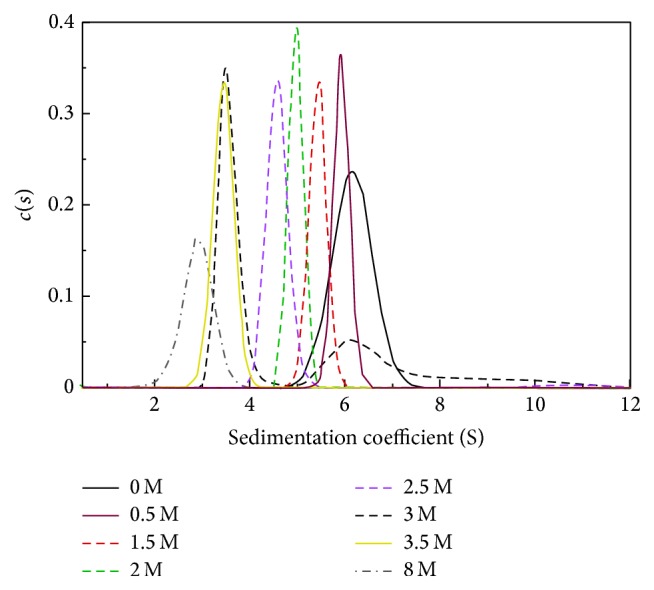
Sedimentation coefficient distribution of WT-alaRS (4 *μ*M) in native form and in the presence of different concentrations of urea. SEDFIT program was used to fit sedimentation velocity data. Considering continuous *c*(*s*) distribution model was considered for the fitting of data. The experiment was performed at 28,000 rpm centrifugation speed at 20°C.

When an oligomeric protein unfolds in the presence of denaturants formation of lower oligomers due to loss of interfacial interactions is possible. It is also interesting to identify the exact nature of this particular state that forms in the denaturation pathway of oligomeric proteins. To understand this in more detail, we had determined the sedimentation coefficients at different urea concentartions.* E. coli* alaRS exists as a dimer in the absence of any denatutants with a sedimentation coefficient of ~6.1 S, consistent with the previous report as dimer [9, 29]. In the presence of 8 M urea, sedimentation value of ~3 S was obtained, which corresposponds to the presesnce of only monomeric species. Remerkably at 3 M urea concentration, the sedimentation data indicated existence of two species, one with a value of ~6 S and the other one with ~3.9 S, confirming the presence of both dimeric and monomeric species. However surprisingly, at 2.5 M and 3.5 M of urea, WT-alaRS exists exclusively as a dimer and a monomer, respectively, as depicted in Figure 7.

## 4. Discussion

*E. coli* alaRS is a large multidomain homodimeric protein whose structure-function relationships are not fully understood. Available biochemical and genetic mutational investigations [18, 32, 33] are insufficient to provide satisfactory information about interactions among its subunits as well as the intrasegmental and domain interactions in its native conformation. Denaturation studies provide valuable information about the nature of forces contributing to proper folding of a protein as well as to its proper functioning. Previously, many folding studies were conducted on oligomeric proteins to understand their conformational stability [34–38]. However, not much information is available on the stability of* E. coli* alaRS. It was reported recently from our group that the full-length alaRS forms an inactive dimeric molten globule-like intermediate in the presence of low concentration of guanidinium hydrochloride [26]. Though both urea and guanidinium hydrochloride have a denaturing effect on proteins, urea has only chaotropic effect, whereas guanidinium hydrochloride has both the ionic and chaotropic effects [39]. Owing to this difference in their mechanism of action, different proteins behave differently in the presence of these two denaturants, as evident from several earlier studies [40–44]. It was established that the oligomerization of alaRS depends on the interaction between the C-terminal coiled-coil domains and a deletion of the C-terminal 175 amino acid produces a mutant monomeric truncated protein (amino acid 1–700) that has the same aminoacylation activity as the wild type protein [32]. These results clearly indicated that alaRS does not need its dimeric structure for aminoacylation; N-terminal part (N-terminal 700 amino acid) of this protein is capable of doing that accurately. To understand the significance of the C-terminal domain in folding and stability, if any, we studied urea induced unfolding pathway of WT-alaRS and its monomeric mutants. The first mutant (G674D) was constructed to provide the information about the contribution of dimerization on the stability of the full-length alaRS. The second mutant was constructed to highlight the role of C-terminal residues (701–875) on the stability of full-length monomeric alaRS. The N461 mutant provides information about the stability of the minimum fragment of the alaRS protein that is needed for the correct aminoacylation of tRNA^Ala^.

Intrinsic tryptophan fluorescence (tryptophan emission maximum shift and the *F*_340_/*F*_350_ ratio) has indicated the presence of an intermediate in the denaturation pathway of wild type alaRS and all its monomeric mutants. The data, when fitted into a three-state equation, yielded the thermodynamic parameters as shown in Table 1. The Δ*G*_total_(Δ*G*_1_ + Δ*G*_2_) of WT-alaRS being 18.6 Kcal/mol is higher than the rest of the mutant proteins that shows similar Δ*G* values (G674D—13.6 Kcal/mol, N700—14.6, and N461—16.7 Kcal/mol). ANS binding studies also indicated the probability of formation of an intermediate in all the alaRS proteins except G674D alaRS. However, far-UV CD experiment ruled out the possibility of formation of an intermediate in the unfolding pathway of all investigated mutant alaRS proteins. When the far-UV CD data (mean residue ellipticity at 222 nm) of monomeric alaRSs (G674D, N700, and N461) were plotted with increasing denaturant concentration the data fitted well in a two-state equation, whereas the WT-alaRS still produces a good fit with a three-state equation. This finding was not unusual considering the fact that far-UV CD signals provide the information of the secondary structures of the entire protein molecule. In contrast, the fluorescence data corresponds to the changes in the microenvironment of tryptophan residues; that is, it provides information about the local or tertiary environment [45]. In WT-alaRS, all the tryptophans are clustered in the N-terminal part of the protein (within the first 461 amino acids) except one; thus the possibility of obtaining similar pattern of fluorescence transitions for all the alaRS proteins is not surprising.

Sedimentation velocity experiments proved that, at 3 M urea,* E. coli* alaRS exists as a mixture of monomeric and dimeric species. Surprisingly tertiary interactions were almost completely lost as indicated by the near-UV CD spectrum at 3 M urea concentration for all the alaRS proteins including WT-alaRS. In a previous report our group has demonstrated that WT-alaRS lost its tertiary structure before the dissolution of the quaternary structure [27]. Similarly, in this study, it was clearly evident from the sedimentation velocity and near-UV CD that at 3 M urea WT-alaRS has lost its tertiary interaction whereas, at this denaturant concentration, some of its quaternary interaction was still present.

When the protein was refolded from the 8 M to 0.8 M urea or lower denaturant concentration and the aminoacylation assay was carried out, the mutant proteins were unable to regain its native conformation as evident from their loss of activity. In contrast, WT-alaRS was able to regain its native-like activity after refolding. The finding that oligomerization provides stability to a protein is not very uncommon. For example, a study conducted on soybean agglutinin has shown that oligomerization imparts a greater stability to the native structure of this legume lectins [46]. Comparison of the folding mechanism of these mutant proteins with the WT-alaRS revealed the role of dimerization in the stability of the native dimeric structure of the protein. This stability to the protein may be conferred by a specific recognition at the dimeric interface essential for folding or due to probable long-range or quaternary interactions. From this study, it was evident that dimerization of alaRS endows the protein with a great deal of conformational stability.

## 5. Conclusion

The present study indicates that the urea induced denaturation of WT-alaRS is a two-state process with no true intermediate being formed. Also the higher stability of the full-length dimeric protein indicates that the C-terminal part plays an important role in the stability of the entire protein.

## Figures and Tables

**Table 1:** The thermodynamic parameters derived from the urea induced denaturation of WT-alaRS, G674D, N700, and N461 alaRS using the data of fluorescence emission maximum. A three-state equation was used to fit the data (equation (2)) as described in Section 2 using the graph plotting software KyPlot (version 2.0 beta 15 (32 bits), Koichi Yoshioka, 1997–2001). The excitation wavelength was kept at 295 nm and emission spectra were recorded from 310 to 450 nm. The emission maximum value was calculated by taking the first derivative of the spectra. Bandpasses for both excitation and emission were kept at 5 nm.

Fluorescence	Δ*G*_1_ (Kcal/mol)	Δ*G*_2_ (Kcal/mol)	*m*_1_ (Kcal/mol/M)	*m*_2_ (Kcal/mol/M)
WT	6.9 ± 0.2^*∗*^	11.7 ± 0.3^*∗*^	1.3 ± 0.1^*∗*^	1.8 ± 0.1^*∗*^
G674D	4.7 ± 0.1^*∗*^	8.9 ± 1.1^*∗*^	0.9 ± 0.1^*∗*^	1.2 ± 0.2^*∗*^
N700	5.9 ± 0.1^*∗*^	8.7 ± 0.2^*∗*^	1.1 ± 0.1^*∗*^	1.3 ± 0.2^*∗*^
N461	3.5 ± 0.2^*∗*^	13.2 ± 0.3^*∗*^	0.8 ± 0.1^*∗*^	1.6 ± 0.1^*∗*^

^*∗*^Values are the means of at least three independent experiments with standard errors indicated.

**Table 2:** Thermodynamic parameters as obtained by fitting the far-UV CD data of WT-alaRS and its variants. The data was fitted in two-state (equation (1)) and three-state equation (equation (2)) for monomeric variants and WT-alaRS, respectively, using the software KyPlot (version 2.0 beta 15 (32 bits), Koichi Yoshioka, 1997–2001). The spectra were recorded in a 1 mm path length cuvette and within a region of 210–260 nm keeping the speed of the scan at 50 nm/min. For averaging, data from five independent scans were taken.

CD	Δ*G*_1_ (Kcal/mol)	Δ*G*_2_ (Kcal/mol)	*m*_1_ (Kcal/mol/M)	*m*_2_ (Kcal/mol/M)
WT	3.2 ± 0.1^*∗*^	13.9 ± 0.1^*∗*^	0.7 ± 0.1^*∗*^	1.7 ± 0.1^*∗*^
G674D	2.7 ± 0.2^*∗*^	—	0.7 ± 0.2^*∗*^	—
N700	2.6 ± 0.3^*∗*^	—	0.6 ± 0.2^*∗*^	—
N461	4.38 ± 0.3^*∗*^	—	0.8 ± 0.1^*∗*^	—

^*∗*^Values are the means of at least three independent experiments with standard errors indicated.

## Abbreviations

aaRS: Aminoacyl-tRNA synthetase
alaRS: Alanyl-tRNA synthetase
ANS: 1-Anilino-8-naphthalene-sulfonate
aa: Amino acid
AUC: Analytical ultracentrifugation
CD: Circular dichroism
SDS: Sodium dodecyl sulphate.
